# The Strategies and Mechanisms of Immune Checkpoint Inhibitors for Brain Metastases in NSCLC

**DOI:** 10.3389/fphar.2022.841623

**Published:** 2022-05-17

**Authors:** Ji Li, Min Wang, Shuhui Xu, Yuying Li, Jiatong Li, Jinming Yu, Hui Zhu

**Affiliations:** ^1^ Department of Oncology, Renmin Hospital of Wuhan University, Wuhan, China; ^2^ Department of Radiation Oncology, Shandong Cancer Hospital and Institute, Shandong First Medical University and Shandong Academy of Medical Sciences, Jinan, China; ^3^ Department of Radiation Oncology, Shandong Cancer Hospital and Institute Affiliated to Shandong University, Jinan, China

**Keywords:** NSCLC, brain metastasis, immune checkpoint inhibitors, irAEs, mechanisms

## Abstract

Brain metastases are more and more common among patients with non-small cell lung cancer (NSCLC). TKI therapy could provide ideal outcomes for patients harboring epidermal growth factor receptor or ALK mutations. For wild-type patients, however, survival is poor because there are few effective treatments other than radiotherapy. Immune checkpoint inhibitors (ICIs) have changed the management of advanced NSCLC. However, the exclusion of patients with active brain metastasis (BM) from most ICI trials precludes the generalization of results. Accordingly, a variety of appropriate real-world studies and clinical trials are being developed to evaluate tumor response. Increasingly encouraging results have suggested that ICIs could be active in the central nervous system (CNS) in select patients with high PD-L1 expression and low CNS disease burden. With the extensive use of ICIs in NSCLC patients with BM, many important questions have emerged concerning issues such as the clinical response to a single ICI, use of ICIs combined with chemotherapy or radiation, the biological mechanism and appropriate sequencing of local and systemic therapy combinations, and safety and toxicity. The present review summarizes the advances in systemic ICIs for the treatment of NSCLC patients with BM, discusses factors associated with efficacy and toxicity, and explores future directions.

## 1 Introduction

Brain metastases (BMs) are common among patients with lung cancer, and the occurrence of BMs has steadily increased with the prolonged survival time. Central nervous system (CNS) metastases, including BMs and leptomeningeal metastases, are present in approximately 10%–20% of non-small cell lung cancer (NSCLC) patients at initial diagnosis. It has been postulated that approximately 25%–40% of NSCLC patients will develop a BM during the course of the disease (Metro et al., 2015). Emerging evidence suggests that patients with epidermal growth factor receptor (EGFR) gene mutation–positive NSCLC are particularly prone to BMs, with the frequency of patients with BM ranging from 44% to 63% (Bhatt et al., 2013). For patients harboring *EGFR* or *ALK* mutations, TKIs have a powerful ability to penetrate the blood–brain barrier and a high potency regarding controlling BM.

For patients without driver mutations, radiation therapy, including stereotactic radiosurgery or stereotactic fractionated radiotherapy (RT), usually combined with systemic chemotherapy, is the main treatment modality. The median survival of NSCLC patients with BM is 6 months with current treatments, but the results vary depending on tumor histology, disease control, patient age, and initial therapy response.

Immune checkpoint inhibitors (ICIs) have changed the management of advanced NSCLC. However, the exclusion of patients with active BMs from most ICI trials precludes the generalization of results. Accordingly, a variety of appropriate real-world studies and clinical trials are being developed to evaluate tumor response.

ICI-induced neurotoxicity is a highly relevant issue because these compounds can enhance immune responses, not only against the tumor but also against self-antigens (McFaline-Figueroa and Lee, 2021). The most common mechanism of neurotoxicity is autoimmunity, and hypophysitis is the most common complication following ipilimumab treatment (up to 17% of patients) (McFaline-Figueroa and Lee, 2021). Conversely, neurologic complications associated with antiPD1 or anti–programmed death ligand 1 (PD-L1) antibody treatment are rare but include acute or chronic inflammatory demyelinating polyneuropathy, myasthenia gravis, and polymyositis. Dual blockade using antiCTLA4 and antiPD1 or anti–PD-L1 antibodies appears to increase the risk of brain edema and headache, but whether it also increases the risk of major immune neurological complications is unclear (Tran et al., 2019; McFaline-Figueroa and Lee, 2021).

In this review, we provide an overview of recent studies using ICIs to treat BMs and discuss how these results are challenging previous paradigms and current clinical practice. Our purpose is to critically summarize advances to date regarding the role and acceptable toxicity of systemic ICIs for the treatment of NSCLC patients with BMs. We also discuss the biological mechanism of systemic therapy combinations. The advances we describe are expected to change the management of NSCLC patients, but further research is needed to address concerns regarding the appropriate sequencing of local and systemic therapy combinations.

## 2 Clinical Outcomes of ICBs in NSCLC With BMs

### 2.1 Immunotherapy Monotherapy

Advanced NSCLC patients with PD-L1–positive (PD-L1 TPS ≥1%) were enrolled in KEYNOTE-001 (NCT01295827), KEYNOTE-010 (NCT01905657), KEYNOTE-024 (NCT02142738), and KEYNOTE-042 (NCT02220894) and then assigned to single pembrolizumab or docetaxel groups. Hazard ratios (HRs) for overall survival (OS) and progression-free survival (PFS) were <1.0 for pembrolizumab vs. chemotherapy, irrespective of baseline BM status. These results showed that pembrolizumab could provide clinical benefit for patients with BMs, such that it has become the standard treatment in patients with advanced PD-L1–positive NSCLC, irrespective of BM status at baseline (Reck et al., 2016; Mansfield et al., 2019).

One prospective phase II trial with pembrolizumab specifically addressed the efficacy of ICIs in patients with BMs, reporting a 29.4% intracranial objective response rate (ORR) in the PD-L1–positive cohort, which was similar to the extracranial ORR (Goldberg et al., 2016). To date, the most robust evidence on the activity of pembrolizumab for the treatment of NSCLC BMs comes from a phase II trial (NCT02085070) that included melanoma and NSCLC patients (18 NSCLC patients and 18 melanoma patients). The diameter of BMs was restricted to between 5 and 20 mm, and patients had to be steroid free and neurologically asymptomatic. In the NSCLC arm, the status of PD-L1 in tumor tissue had to be positive (≥1%). Eight of the 18 NSCLC patients had received no prior local therapy for BMs. Six of 18 patients (33%) achieved an intracranial response. Systemic RR was 33%, with only one patient suffering progression in the CNS while responding systemically. All other responses were concordant (Goldberg et al., 2020). The above data showed that pembrolizumab monotherapy could provide excellent intracranial response for patients with PD-L1 ≥1%.

Nivolumab, another anti-PD1 agent, has been affirmed as second-line standard treatment basing on the CheckMate017 and 057 studies. In order to evaluate the efficacy of nivolumab in patients with BMs, a pooled analysis including patients with NSCLC and pretreated stable BMs who were enrolled in three nivolumab clinical trials (CheckMate 063, 017, and 057) was conducted. A total of 46 patients with BMs in the nivolumab group and 42 patients receiving docetaxel as second-line treatment were analyzed. Most patients had been previously treated with brain RT (74% in the nivolumab group and 83% in the docetaxel group). The median time to new BM occurrence and median survival time were 8 and 8.4 months, respectively, in the nivolumab group and 9 and 6.2 months, respectively, in the docetaxel group (Antonia et al., 2019).

Another retrospective study enrolled 5 patients with asymptomatic and corticosteroid-free BMs before nivolumab initiation. Two intracranial responses were observed, which were maintained for up to 24 and 28 weeks. Notably, intracranial and systemic responses were largely concordant, except in one patient in whom stable CNS disease was associated with rapid systemic progression (Dudnik et al., 2016). Real-world data from 38 patients with squamous NSCLC and asymptomatic BM were also published. One patient obtained CR, 6 patients achieved partial response (PR), and 11 achieved stable disease after nivolumab treatment (Bidoli et al., 2016). In an Italian clinical trial, 409 NSCLC BM patients were treated with nivolumab. The Kaplan-Meier method was used to estimate PFS and OS. Overall RR was 17% in patients with BMs and 18% in the entire cohort. Median PFS and OS were 3 and 8.6 months in patients with BMs and 3 and 11.3 months in the entire cohort, respectively (Crinò et al., 2019). These above trials showed that nivolumab also provides clinical benefit for NSCLC with BMs.

With regard to the activity of anti–PD-L1 ICI therapy in patients with BMs, the OAK trial evaluated the efficacy in the BM subgroup. The results showed that atezolizumab was superior to docetaxel in terms of delaying the appearance of new symptomatic BMs (HR 0.38, 95% confidence interval [CI]: 0.16–0.91) (Rittmeyer et al., 2017; Fehrenbacher et al., 2018; Gadgeel et al., 2019).

Some reports, however, have suggested that the RR of BMs in real-world settings might be lower than in more-selective clinical trial populations (Kim et al., 2019). Hendriks showed that the ORRs were similar: 20.6% in the BM group and 22.7% in non-BM group. However, the disease control rate (DCR) was significantly lower in patients with BMs, 43.9% vs. 52.0%. The median PFS was 1.7 and 2.1 months in the BM and non-BM groups, respectively. The median OS of patients with BMs was 8.6 months, compared with 11.4 months for patients with no BMs (*p* = 0.035). In the BM subgroup multivariate analysis, stable BMs and higher diagnosis-specific graded prognostic assessment (DS-GPA) classification were associated with improved OS (Hendriks et al., 2019).

The safety of ICIs alone in patients with BMs has been reported in several pooled analyses including data from five clinical trials. These studies showed that among 1,452 patients, serious neurological AE incidence was higher in patients with BMs compared to those without BMs (6% vs. 3%), but none reached grade 4. Thus, the abovementioned results suggest that PD-L1–positive NSCLC patients with BMs could benefit more from treatment with ICIs, with no relevant serious safety differences reported (Lukas et al., 2017). Clinical trials of immunotherapy monotherapy for NSCLC with BMs are detailed in Table 1.

**TABLE 1:** Clinical investigations of Immunotherapy monotherapy in the treatment of NSCLC patients with brain metastases.

Study	Number	Line	ICI arm vs. control arm	PD-L1 status	Number of patients with BM included	Brain metastasis inclusion criteria	ORR, PFS, OS	Toxicities
Checkmate 026	NCT02041533 Kluger et al. (2019)	First-line	Nivolumab vs. platinum doublet	≥1%	69 (13%)	Pretreated, off corticosteroids or on a stable or decreasing dose of ≤10 mg daily prednisone and stable	NA	Grade 3 or 4:18%
	NCT02085070 Goldberg et al. (2020)	Second-line	Pembrolizumab	≥1%	18	BM 5–20 mm	ORR was 33%	Neurologic AEs:all grade ≤2
Keynote 024	NCT02142738 Reck et al. (2019)	First-line	Pembrolizumab vs. platinum doublet	≥50%	28 (9.1%)	Pretreated, off corticosteroids and stable	PFS 0.55 (0.20–1.56)	grade 3 to 5 adverse events:(31.2% v 53.3%)
							OS 0.73 (0.20–2.62)	
Keynote 042	NCT02220894 Mok et al. (2019)	First-line	Pembrolizumab vs. platinum doublet	≥1%	70 (5.5%)	Pretreated, off corticosteroids and stable	NA	grade 3 to 5 adverse events:(18% v 41%)
Keynote 010	Herbst et al. (2016)	Second-line NSCLC	Pembrolizumab vs. docetaxel	≥1%	152 (14.7%)	Pretreated, off corticosteroids and stable	NA	grade 3 to 5 adverse events:(13% v 35%)
Checkmate017	Brahmer et al. (2015), Goldman et al. (2016)	Second-line Squamous carcinoma	Nivolumab vs. docetaxel	All comers	17 (6%)	Pretreated, off corticosteroids or on a stable or decreasing dose of ≤10 mg daily prednisone and stable	5.0 vs. 3.86 m HR NA	CNS trAEs: 7%
Checkmate057	Brahmer et al. (2015), Goldman et al. (2016)	Second-line Non-squamous carcinoma	Nivolumab vs. docetaxel	All comers	68 (12%)	Pretreated, off corticosteroids or on a stable or decreasing dose of ≤10 mg daily prednisone and stable	7.6 vs. 7.3 m HR 1.04 (0.62–1.76)	trAEs: 0%
OAK研究	Rittmeyer et al. (2017), Mansfield et al. (2019)	Second-line NSCLC	Atezolizumab vs. docetaxel	All comers	123 (10%)	Pretreated, off corticosteroids, stable and supratentorial	OS 16.0 M 0.74 (0.49–1.13)	NR
							PFS 0.38 (0.16–0.91)	

**FIGURE 1:**
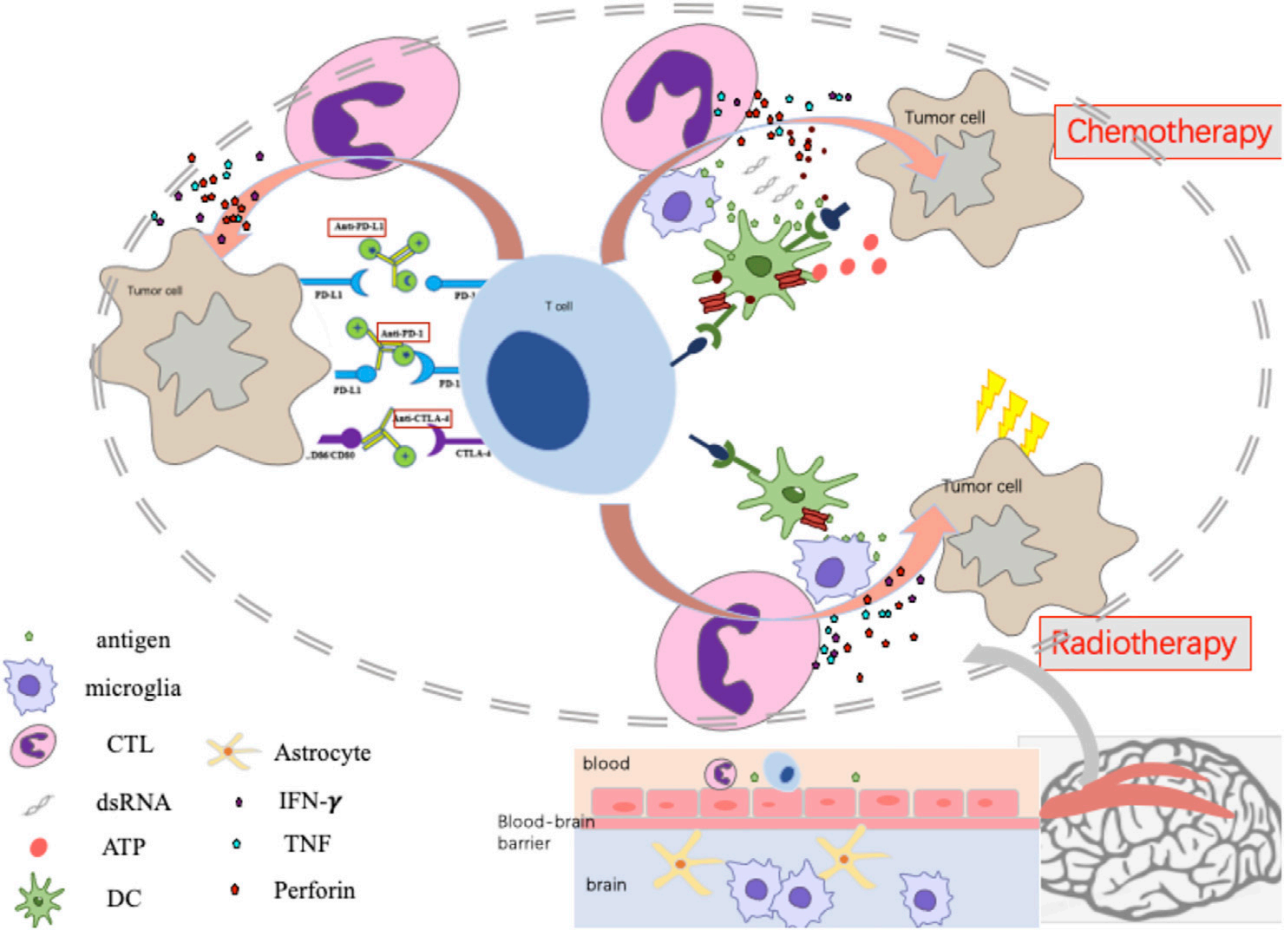
The mechanism of Immunotherapy monotherapy, Immune-checkpoint inhibitor combined with Chemotherapy or Radiotherapy.

These data demonstrate that the efficacy of ICIs alone in patients with NSCLC with BMs is limited. ICI therapy combined with other treatments such as chemotherapy, radiation therapy, and anti-angiogenic therapy may be more clinically advantageous for this special subgroup. The possible mechanism and pathway of ICI combined modalities are summarized in Figure 1. The mechanism of combined modalities and their clinical outcomes are discussed below.

### 2.2 ICI Therapy Combined With Chemotherapy

Chemotherapy is known to promote tumor immunity in two major ways, *via* inducing immunogenic cell death and disrupting the ability of tumors to evade the immune response. This process involves the concomitant release of tumor antigens during cell death. Anthracyclines activate expression of the pattern recognition receptor Toll-like receptor-3, the rapid secretion of type I interferons (IFNs), and release of the chemokine CXCL10. The type I IFN gene signature predicts the response to anthracycline therapy in breast cancer patients (Sistigu et al., 2014). Phylogenetically conserved chemokine signaling *via* CXCL8 increases the exposure of calreticulin on the tumor cell surface, which is critical for dendritic cells (DCs) to recognize and engulf dying tumor cells (Sukkurwala et al., 2014). Immunogenic chemotherapy–induced cell death also induces autophagy (Michaud et al., 2011) and necroptosis (Inoue and Tani, 2014). In addition to inducing immunogenic cell death and type I IFN secretion, anthracyclines promote the recruitment of CCL2/CCR2–dependent functional antigen-presenting cells (APCs) into tumor sites but not into tumor-draining lymph nodes (Ma et al., 2014). Chemotherapy can enhance tumor antigen presentation by either upregulating the expression of tumor antigens themselves or antigen-bound MHC class I molecules. Alternatively, chemotherapy can upregulate the expression of costimulatory molecules (B7-1) or downregulate the expression of co-inhibitory molecules (PD-L1/B7-H1 or B7-H4) expressed on the tumor cell surface, enhancing the activity of effector T cells (Wargo et al., 2015). Therefore, treatment strategies combining and ICI with chemotherapy may have certain application value in the treatment of NSCLC BMs, but the choice of specific regimen needs further study.

The KEYNOTE series clinical trials (021, 189, and 407) showed that pembrolizumab combined with chemotherapy provides longer survival than chemotherapy alone, irrespective of the presence of BMs at baseline (Powell et al., 2019). In the whole study group, 13% (171/1298) had baseline BMs. Median (range) follow-up was 10.9 (0.1–35.1) months and 11.0 (0.1–34.9) months in patients with and without BMs, respectively. Among patients with BMs, pembrolizumab plus chemotherapy had a median OS of 18.8 months, chemotherapy had a median OS of 7.6 months, and median PFS was 6.9 and 4.1 months, respectively. In the patients with BMs, grade 3–5 AEs were observed in 81.4% of patients in the pembrolizumab plus chemotherapy group and 70.3% in the chemotherapy alone group. In patients without BMs, the rate of AEs was lower than in patients with BMs: 68.3% and 65.6% in pembrolizumab plus chemotherapy group and chemotherapy alone group, respectively. Thus, pembrolizumab plus chemotherapy provides clinical benefit for patients with NSCLC, regardless of the presence/absence of stable BMs (Powell et al., 2019). The KEYNOTE-189 trial included some patients with previously treated stable and untreated asymptomatic BMs (with no lesions larger than 1.5 cm). It was recently reported that the benefit of the combination in terms of PFS and OS was confirmed in the subgroup of patients with BMs: HR (95% CI) of 0.42 (0.27–0.67) and 0.41 (0.24–0.67), respectively. Notably, the magnitude of benefit attributable to the addition of pembrolizumab was greater in patients with BMs than in those without CNS involvement (Gandhi et al., 2018). A real-world retrospective cohort experience with the same combination of drugs reported similar results (Afzal et al., 2018). The results of clinical trials of ICI combined with chemotherapy for NSCLC with BMs are detailed in Table 2.

**TABLE 2:** Clinical investigations of ICI combined with chemotherapy in the treatment of NSCLC patients with brain metastases.

Study	Number	Line	ICI arm vs. control arm	PD-L1 status	Number of patients with BM included	Brain metastasis inclusion criteria	ORR, PFS, OS	Toxicities
Keynote 189	NCT02578680 Gandhi et al. (2018)	First-line Non-squamous carcinoma	Carboplatin-pemetrexed + pembrolizumab vs. Carboplatin-pemetrexed + placebo	All comers	108 (17.5%)	Previously treated, stable and off corticosteroids or untreated, asymptomatic and off corticosteroids	OS 19.2M 0.42 (0.27–0.67)	grade 3 to 5 adverse events:(67.2% v 65.8%)
							PFS 6.9M 0.41 (0.24–0.67)	
Keynote 407	NCT02775435 Paz-Ares et al. (2018)	First-line Squamous carcinoma	Carboplatin-(nab)paclitaxel + pembrolizumab vs. Carboplatin-(nab)paclitaxel + placebo	All comers	44 (7.8%)	Previously treated, stable and off corticosteroids or untreated, asymptomatic and off corticosteroids	NA	grade 3 to 5 adverse events:(13.3% v 6.4%)
Impower 130	NCT02367781 West et al. (2019)	Non-squamous carcinoma	Carboplatin + nab-paclitaxel + atezolizumab vs. carboplatin + nab-paclitaxel	All comers	NA	Pretreated, off corticosteroids, stable, supratentorial or cerebellar	NA	NA
Impower 131	NCT02367794 (Jotte et al., 2019)	Squamous carcinoma	Atezolizumab + carboplatin-(nab)paclitaxel vs. carboplatin-(nab)paclitaxel	All comers	NA	Pretreated, off corticosteroids, stable, supratentorial or cerebellar	NA	grade 3 OR 4 adverse events:(68% v 21%)
Impower 132	NCT02657434 Papadimitrakopoulou et al. (2018)	Non-squamous carcinoma	Platinum-pemetrexed + atezolizumab vs. platinum-pemetrexed	All comers	NA	Pretreated, off corticosteroids, stable, supratentorial or cerebellar	NA	grade 3 OR 4 adverse events:(11.3% v 10.1%)

### 2.3 Combination ICI and RT

RT induces the immunogenic death of cancer cells, which is characterized by the transfer of calreticulin to the surface of dying tumor cells together with high-mobility group box-1 and adenosine triphosphate release. The dying cells release danger-associated molecular patterns (DAMPs) and promote the transfer of tumour-associated antigens to dendritic cells (DCs), and thus activate DC maturation (Sistigu et al., 2014). RT synergistically promotes tumor antigen uptake and cross-presentation by DCs to T cells in the draining lymph nodes. Radiation can prompt a rapid increase in the number of tumor-infiltrating CD8^+^ T cells. Tumor-derived DNA activates DNA-sensing pathways to induce IFNβ production by DCs, which is required for DC activation. Simultaneously, activation of transforming growth factor β (TGFβ) and colony-stimulating factor 1 increases infiltration of T regulatory cells (Tregs) and myeloid-derived suppressor cells (MDSCs) (DemariaGolden and Formenti, 2015). From the perspective of the abscopal effect, the migration of T cells primed against TAA could enhance the anti-tumor immune response to other lesions with similar antigens, and multi-focal RT may promote this effect more effectively (Brooks and Chang, 2019). In addition, one study suggested that for NSCLC patients with negative PD-L1 expression in the primary origin, RT may lead to positive changes in BMs (Takamori et al., 2017). In summary, RT can affect immunotherapy through a variety of pathways.

There is a strong rationale behind the combination of RT and ICI, both preclinical and clinical. The rationale behind the combination originally stemmed from the observation of the abscopal effect. The abscopal effect is a phenomenon in which radiation at one site leads to the regression of metastatic cancer at a distant site that has not been exposed to any radiation. Before the era of clinical cancer immunotherapy, anecdotal reports of the abscopal effect suggested that the response of tumors distant from the irradiation field could be immune-mediated (Abuodeh et al., 2016). This hypothesis was supported by loss- and gain-of-function experiments using T-cell–deficient athymic nude mice and DC enhancers, respectively (Demaria et al., 2004). Some preliminary reports suggested that immune enhancers could significantly contribute to abscopal responses in patients with a variety of solid cancers (Golden et al., 2015), but confirmatory results for combining modern ICI and RT on BM are still awaited. Moreover, additional complexity is suggested by trials in which preclinical and clinical data suggest that appropriate timing and dosing of irradiation might be critical for the induction of an effective anti-tumor immune response (Vanpouille-Box et al., 2017; Minniti et al., 2019).

Synergism or additivity of ICI combined with RT can be assessed in the CNS. Increased PD-L1 expression in resected BM specimens after radiation supports this potential interplay (Takamori et al., 2018). Interestingly, a variety of retrospective analyses from observational studies have suggested that the combination of RT and ICI therapy could have a positive impact on patient survival over exclusively systemic treatment (Shaverdian et al., 2017; Chen et al., 2018; Karivedu et al., 2018).

One retrospective study evaluated concurrent ipilimumab therapy and stereotactic radiosurgery (SRS) in 46 patients with 113 BMs. The results showed that SRS during or before treatment with ipilimumab provided better OS and less regional recurrence compared with patients treated with SRS lately (Kiess et al., 2015). Another group analyzed 25 patients with 58 BMs and found increased time to regional brain control and CNS progression when SRS was delivered within 30 days of immunotherapy, compared with patients not treated simultaneously (Skrepnik et al., 2017). A study that analyzed over 560 intracranial lesions in 75 patients with melanoma BMs, provided perhaps the best evidence for the benefit of concurrent therapy. The authors of that study found that concurrent therapy involving SRS and immunotherapy significantly decreased the lesion volume compared with non-concurrent treatment (Qian et al., 2016). Although most of the literature suggests that concurrent therapy is beneficial, it should be noted that no benefit was found in another study involving 35 patients (Liniker et al., 2016). The above data demonstrate that SRS combined with an ICI is a useful modality for patients with BMs.

Some studies have explored which NSCLC patients with BMs could obtain the best benefit from SRS combined with an ICI. Singh et al. reported that Karnofsky performance status (KPS) score <80 (*p* = 0.001) and lung-specific molecular marker graded prognostic assessment (lung molGPA) score <1.5 (*p* = 0.02) were the inferior predictors of OS. There was no significant benefit in terms of either OS or total degree of lesional response in patients with both KPS score <80 and lung molGPA score <1.5 when anti–PD-1 was combined with SRS. However, in lesions with a volume >500 mm^3^, combining SRS with an ICI resulted in faster and better volumetric response, which was particularly beneficial for patients with mass effects (Singh et al., 2019).

Recently, the fraction of radiation was evaluated to elucidate the relationship of them and AEs in a retrospective single-center analysis. Of 163 patients, 50 (31%) patients received an ICI, while 113 (69%) were ICI-naïve. Overall, 94 (58%), 28 (17%), and 101 (62%) patients received SRS, partial brain irradiation, and/or whole-brain RT (WBRT), respectively. Fifty percent of patient received more than one radiation course. No significant differences in rates of all-grade AEs or grade ≥3 AEs between ICI-naïve and ICI-treated patients were observed across different cranial RT types (grade ≥3 AEs: 8% ICI− vs. 9% ICI + for SRS [*p* = 1.00]; 8% ICI− vs. 10% ICI + for WBRT [*p* = 0.71]). Additionally, there were no differences in AE rates based on the timing of ICI administration with respect to RT (Ahmed et al., 2017). However, a small-sample study found an association between receipt of immunotherapy and symptomatic radiation necrosis (RN) in patients with BMs undergoing stereotactic radiation (HR: 2.56; 95% CI: 1.35–4.86; *p* = 0.004) (Nimmerjahn et al., 2005a).

Another retrospective study from a small series supported the hypothesis that the neurological toxicity of an ICI combined with brain RT is manageable, with no patients undergoing surgical resection for symptomatic RN among 17 patients who received SRT and nivolumab or durvalumab (Martin et al., 2018a). However, a recently published retrospective evaluation showed that, treatment with an ICI was associated with a significantly increased risk of symptomatic RN, regardless of tumor histology. Notably, a tendency toward increased symptomatic RN remained, but statistical significance was not reached if patients who received ipilimumab were excluded from the analysis (Kluger et al., 2019). Of note, the median time to RN after SRT in patients treated with immunotherapy was about 10 months. Therefore, the patients with prolonged survival could have biased the increased RN incidence. An innovative clinical trial (NCT02681549) that is currently recruiting patients with melanoma and NSCLC BMs intends to evaluate whether bevacizumab in combination with pembrolizumab is capable of reducing brain edema and RN incidence while potentially synergizing with immune cell trafficking (Colaco et al., 2016; Kluger et al., 2019).

### 2.4 Combination of ICI and Anti-Angiogenic Drugs

The growth and metastasis of a tumor depends on the formation of blood vessels for nutrition, and anti-angiogenic therapy can reduce the oxygen content in tumors and thereby delay tumor growth. Normalization of blood vessels is beneficial for sensitization to chemotherapy and RT. It can also reduce cerebral edema in patients with BMs. In addition, anti-angiogenic therapy is performed by inhibiting endothelial cell proliferation, thus eliminating the need to cross the blood-brain barrier (BBB) (Carbone et al., 2017).

Vascular endothelial growth factor (VEGF) exerts a systemic effect on immune-regulatory cell function *via* multiple mechanisms. VEGF induces the proliferation of inhibitory immune-cell subsets, such as Tregs and MDSCs, inhibits DC maturation, and inhibits T-cell development from hematopoietic progenitor cells (Gabrilovich et al., 1998; Shojaei et al., 2007). MDSCs also secret angiogenic factors such as *Bombina* variegata peptide 8 and VEGF, upregulating STAT3 to promote tumor metastasis (Feng et al., 2018). Therefore, anti-VEGF antibodies may promote the normalization of tumor blood vessels, reshaping the tumor microenvironment and enhancing the effect of ICIs (Chongsathidkiet et al., 2019). Immunotherapy combined with anti-angiogenic therapy has complementary effects: anti-angiogenic therapy plays a role in antigen recognition, immune cell recruitment, and remodeling of the immune microenvironment, whereas immunotherapy can restore immune function (Khan and Kerbel, 2018).

In recent years, the safety and efficacy of bevacizumab as an anti-angiogenic agent in the treatment of NSCLC with BMs have been recognized (Socinski et al., 2018). The results of a phase III clinical trial, IMPOWER 150, were presented at ASCO 2020. This study demonstrate that, compared with ACP (atezolizumab plus carboplatin plus paclitaxel) group, the incidence of new BMs was lower in the atezolizumab plus bevacizumab plus carboplatin plus paclitaxel (ABCP) and bevacizumab plus carboplatin plus paclitaxel (BCP) groups that received bevacizumab, and the results were similar between the two groups (7% and 6%). A tendency toward delayed onset of BM was observed in the ABCP group. The study’s data suggested that the addition of atezolizumab to BCP may not reduce the incidence of new BMs, but may delay TTD (time to development). In conclusion, bevacizumab, especially when combined with an ICI, plays a definite role in delaying the occurrence of BMs in patients with metastatic non-squamous NSCLC (Federico et al., 2020). The international, randomized, double-blind phase III study, ONO-4538-52/TASUKI-52, evaluated nivolumab with bevacizumab and cytotoxic chemotherapy as first-line treatment for nonsquamous non-small-cell lung cancer (NSCLC). Among patients with BMs subgroup, nivolumab arm had a median PFS of 10.6 months, placebo arm had a median PFS of 7.1 months. Potential benefits have been observed in patients with BMs, but these results need to be confirmed by prospective studies (D’après Lee, 2020).

### 2.5 Combination ICIs

Combination ICI therapy, such as the combination of anti–CTLA-4 and anti–PD-1, aims to recruit T cells into tumors and then prevent them from being “turned off.” CTLA4 blockade induces frequent increases in intratumoral T cell infiltration beyond its clinical response rate (Zhou et al., 2018a), and concurrent ipilimumab plus nivolumab in melanoma has shown promise (Long et al., 2017; Martin et al., 2018b).

CheckMate 012 was a phase I multi-cohort study evaluating the safety and tolerability of nivolumab alone or in combination with other therapies in patients with advanced NSCLC. The study analyzed 12 patients with at least one asymptomatic and untreated BM. Intracranial responses occurred in 2 patients (iCR: 16.7%; 95% CI: 2.1–48.4); the median PFS was 1.6 months (95% CI: 0.92–2.50), and the median OS was 8.0 months (95% CI: 1.38–15.50). No treatment-related neurological adverse events were reported. The median OS was longer than chemotherapy. Therefore, combined ICI treatment might also result in favorable outcomes for patients with NSCLC and BMs (Goldman et al., 2016; Hellmann et al., 2019; Reck et al., 2020). However, a large-sample clinical trial is needed, and the irAEs need more attention.

## 3 Factors Related to the Efficacy of Immunotherapy for BMs

### 3.1 BBB

**FIGURE 2:**
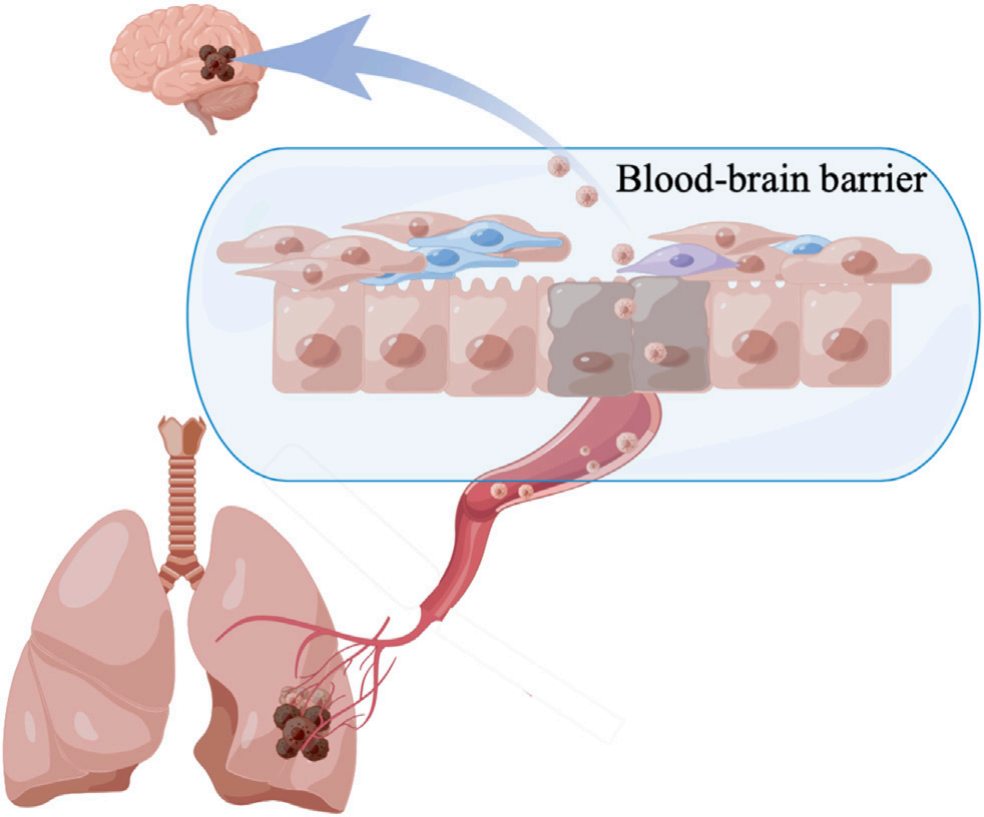
The mechanism of BMs.

Because the immune system in the brain differs from that of the rest of the body, access to the tumors is limited by the BBB, and the host is subject to substantial endogenous and treatment-induced immunosuppression (Figure 2). The BBB is a dynamic interface between the systemic blood circulation and the CNS that selectively blocks the passage of harmful substances to keep them out of the brain tissue. The neurovascular unit is the basic unit of the BBB and includes endothelial cells, pericytes, and astrocytic end-feet (Kim et al., 2016; Iadecola, 2017). Multiple signaling pathways determine the role of the BBB, such as oligodendrocyte precursor cells that reduce BBB leakage *via* the Wnt/β-catenin pathway (Van Steenwinckel et al., 2019; Wang et al., 2020). The sonic hedgehog protein secreted by CNS astrocytes, increases the protein level of netrin-1 to regulate features of the vascular architecture and function (Alvarez et al., 2011; Arvanitis et al., 2020). TGF-β/Smad signaling plays a unique role in maintaining cerebrovascular integrity (Li et al., 2011). In addition, lipopolysaccharide can increase the permeability of endothelial cells (Haileselassie et al., 2020). New research has overturned the idea that the BBB prevents immune cells from entering the brain and demonstrated the entry of T cells and immune monitoring within the brain. Similarly, in the case of gliomas and other tumors, damage to the BBB limits the protection that it normally provides (Owens et al., 2008).

The brain is considered an immune-privileged site due to the presence of the BBB, and this may inhibit the therapeutic efficacy of ICIs. Because ICIs block the inhibitory signals of T-cell activation, T cells are able to respond against tumors. However, due to poor prognosis and risk of pseudoprogression of brain tumors, patients with untreated or active BMs are systematically excluded from clinical trials of ICIs.

### 3.2 PD-L1 Expression in BMs

Activation of the PD-1 pathway is a common mechanism that blunts the killing function of effector T cells by tumor cells and immune cells in their microenvironment, and this is an important pathway for tumor immune escape (termed adaptive immune resistance) (Tumeh et al., 2014). PD-L1 (B7-H1) emerged early as a marker associated with PD-1 inhibition and is widely expressed in different tumor types (Taube et al., 2014). Similar to other metastatic tumors, there is often spatio-temporal heterogeneity in tumor immunity between BMs and their lung primary tumors. Mansfield analyzed 146 paired primary lung cancers and BMs from 73 cases. There was a difference in tumor cell PD-L1 expression in 10 cases (14%) and a difference in TIL PD-L1 expression in 19 cases (26%). These data suggest that heterogeneity between intracranial and extracranial lesions should be considered (Mansfield et al., 2016). Zhou found that in patients with BMs, in addition to 32% of patients expressing PD-L1 on TCs and ICs, patients simultaneously expressing PD-L1 only on TCs or ICs also had different groups (for example, 8% of patients expressed PD-L1 only on ICs). Further analysis of PD-L1 overexpression patterns showed that only 4% of patients with primary lesions exhibited high expression of PD-L1 on both TCs and ICs, whereas 16% of patients with BMs had strong positive expression of PD-L1 on both TCs and ICs (Zhou et al., 2018b).

One pooled analysis from seven European cancer centers included NSCLC patients who were treated with ICIs in a variety of settings, including daily standard practice. Among patients with active BMs that were evaluable in the CNS (*n* = 73), the intracranial RR was 27.3%. Among the 23 patients with active BMs and available PD-L1 expression status, positive expression of PD-L1 (≥1%) was associated with a higher intracranial RR, 35.7% vs. 11.1% in PD-L1–negative patients (Federico et al., 2020). Recently, updated data from 34 patients with PD-L1–positive tumors and 5 patients with PD-L1–negative disease showed that CNS RR was 10/34 (29.4%) in PD-L1–positive patients, with a median duration of response of 10.7 months. Discordance was observed between intracranial and systemic responses in 7 patients. Among these, 4 individuals experienced PD in the brain and PR systemically, whereas the remaining 3 patients exhibited the opposite findings. Interestingly, no intracranial responses were observed among patients with PD-L1–negative tumors (Goldberg et al., 2018).

### 3.3 BM Microenvironment Immunobiology

The CNS is armed with resident myeloid cells such as microglia, which are innate immune cells in the CNS, the brain’s equivalent of tissue-resident macrophages, and perivascular macrophages that colonize the CNS in early development and maintain homeostasis in the brain parenchyma and at brain–blood vessel interfaces (Owens et al., 2008). Microglia and macrophages are different from tumor cells in that they are involved in multiple stages of disease metastasis and are genetically stable and predictable. Under physiological conditions, microglia participate in regulation of the nervous system by phagocytosis and removal of apoptosis debris and do not cause inflammation, thereby contributing to CNS homeostasis (Nimmerjahn et al., 2005b; Glass et al., 2010; Ginhoux et al., 2013). CD49D is considered a good flow cytometry marker for the identification of microglia and peripheral macrophages in human brain tumors (Bowman et al., 2016). Cancer cells increase their proliferation and survival by polarizing microglia and infiltrating surrounding macrophages through a variety of mechanisms (Raza et al., 2018).

CNS tumors exhibit a low number of tumor-infiltrating lymphocytes (TILs) and other immune effector cell types that are different from other tumor types. Although both primary and metastatic brain malignancies can subvert and manipulate immune response, their immune landscapes are of great significance. In general, metastatic brain tumor has higher level of T cells and neutrophils infiltration compared with primary brain tumors including glioblastoma and gliomas, which were considered as “immune-cold” tumors (Klemm et al., 2020; Sampson et al., 2020). Primary brain tumors also have lower TMB compared to brain metastatic tumors including NSCLC and melanomas (Alexandrov et al., 2013). As result, patients with primary brain tumor shows inferior response to ICI treatment compared to NSCLC or melanoma patients with brain metastasis (Tawbi et al., 2018; Cloughesy et al., 2019). This “cold tumor” phenotype is associated with adverse reactions to immune-stimulating therapies, such as immune checkpoint blockade (Gajewski et al., 2017). For decades, brain has been regarded as one of the “immune privileged” organs in body, yet, the discovery of functional lymphatic vessels in meninges in 2015 suggested that the CNS immune privilege paradigm is overstated (Aspelund et al., 2015). However, as a number of mechanisms have been evolved by brain to restrict immune response, which may be harmful, many have suggested that brain should be considered as a “immune distinct” organ (Kipnis, 2016). Even when T-cell responses are induced by means such as vaccination, the number of antigen-specific TILs is still very low, and those that are present often exhibit a depleted phenotype (Keskin et al., 2019). Due to the unique immune microenvironment of the brain, the decrease in the number and activity of T cells in CNS tumors is largely limited. Uncontrolled inflammation in the brain poses a threat that inflammation in peripheral organs does not because of the brain’s rigid outer shell and the potential for damage from elevated intracranial pressure. Thus, the CNS may have evolved into a special immune microenvironment in which both inflammation and adaptive immune responses are tightly regulated. This regulation involves a variety of mechanisms at the molecular and cellular levels (Quail and Joyce, 2017).

**FIGURE 3:**
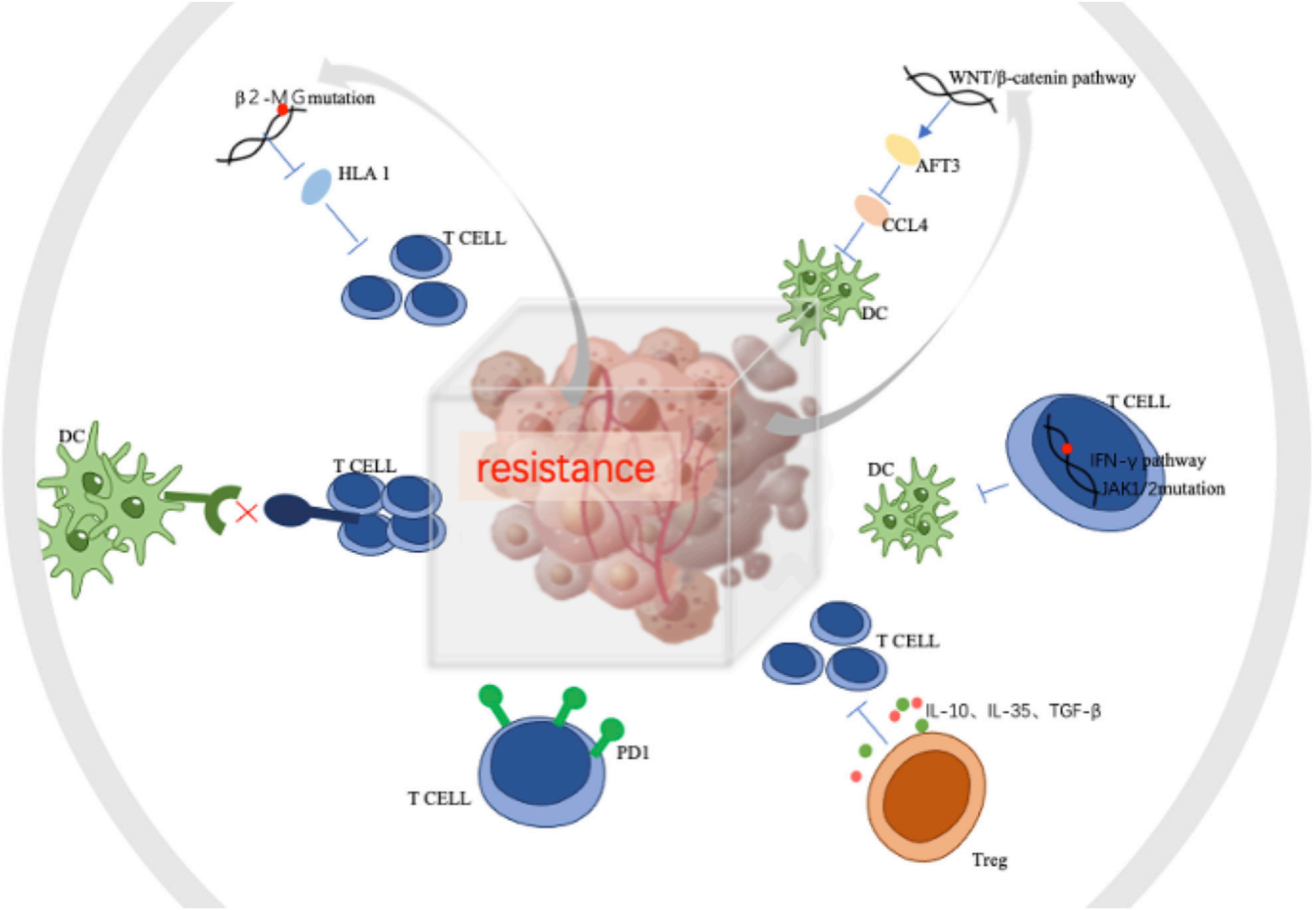
The mechanism of immuno-resistance.

Tumor-associated macrophages (TAMs) and microglia communicate significantly with tumor cells in the brain. Brain tumor cells release cytokines and chemokines to recruit TAMs into the microenvironment, and TAMs in turn provide tumor-promoting and survival factors. DCs can present tumor antigens to T cells to induce an anti-tumor immune response. Increased neutrophils in brain tumor tissue are associated with bevacizumab resistance and the development of high-grade gliomas. Tregs can inhibit cytotoxic T cells, leading to an immunosuppressive microenvironment that allows tumor growth. Astrocytes are unique to the CNS and act as physical channels for signaling molecules in a heterogeneous manner (Quail and Joyce, 2017). The mechanism of immuno-resistance was showen in Figure 3.

## 4 Future Perspectives for NSCLC With BMs

Drug delivery faces major challenges due to the presence of the BBB (Tang et al., 2019). Extracellular vesicles (EVs), including all membrane-derived vesicles located outside the cell, have developed into important intercellular communication agents (Tkach and Théry, 2016). The *in vivo* efficacy of exosome delivery of anticancer drug into the brain was studied in zebrafish embryos (Yang et al., 2015). Moreover, exosomes were capable of delivering siRNA to the brain, across the BBB in mice (Alvarez-Erviti et al., 2011). Many studies have indicated that exosomes can function in innate and adaptive immune responses (Kurywchak et al., 2018). Tumor-derived exosomes and immune-cell-derived exosomes enhance anti-tumor responses by activating antigen-specific T-cell responses by transferring antigens to APCs. Our recent studies have shown that EVs contribute to the progression of a variety of brain diseases, including BM, and they are thus considered promising therapeutic and drug delivery vectors. Many studies have determined that tumor-derived EVs can disrupt the intact BBB *in vivo*. Further clinical studies are needed to investigate the optimal chemotherapy regimens, appropriate doses, and fraction of RT when combined with immunotherapy to treat BMs (Table 3).

**TABLE 3:** Selected ongoing clinical trials of immunotherapy in NSCLC patients with brain metastases.

NCT identifier	Study phase	Treatment	Study population	Primary end point	Status	Enrollment
NCT02681549	II	Pembrolizumab plus bevacizumab	NSCLC, melanoma	Brain metastases response rate	Recruiting	53
NCT02978404	II	Nivolumab plus SRS	NSCLC and SCLC with brain metastases	Intracranial PFS	Recruiting	26
NCT02696993	I/II	Nivolumab and radiotherapy with or without ipilimumab	NSCLC with brain metastases	1) RP2D for nivolumab; 2) RP2D for ipilimumab; 3) intracranial PFS	Recruiting	88
NCT04211090	II	Camrelizumab plus pemetrexed and carboplatin	NSCLC	iORR	Recruiting	64
NCT04213170	II	Sintilimab plus bevacizumab	NSCLC	iORR	Recruiting	60
NCT04507217	II	Tislelizumab plus carboplatin and pemetrexed	NSCLC	OS PFS	Not yet recruiting	78
NCT04333004	I/II	Pembrolizumab plus chemotherapy	NSCLC	iORR iPFS PFS	Not yet recruiting	162
NCT02858869	I	Pembrolizumab plus SRS	NSCLC, Melanoma	iORR	Recruiting	30
NCT03325166	II	Pembrolizumab plus SRS	NSCLC	iORR	Recruiting	20
NCT04434560	II	Ipilimumab plus nivolumab as neoadjuvant therapy	NSCLC, Melanoma, Brest cancer Renal cell carcinoma	Feasibility	Not yet recruiting	40
NCT04291092	II	Camrelizumab plus WBRT	NSCLC	12-week PFS rate	Not yet recruiting	20
NCT04180501	II	Sintilimab plus SRS	NSCLC	iPFS	Recruiting	25

## 5 Conclusion

ICIs are challenging the traditional idea that monoclonal antibodies are marginally active against BMs. There is encouraging preliminary evidence to support the efficiency of ICIs alone or combined with other treatments. Based on current evidence, ICIs play a relevant role in acquisition of long-term disease control in the CNS of patients with advanced NSCLC. Despite the encouraging efficacy of ICIs in treating BMs, other modalities that can enhance the activity of anti–PD-1/PD-L1 monotherapy are urgently needed, and prospective trials in NSCLC BM patients are warranted. Additionally, improved imaging modalities are needed to differentiate RN, pseudoprogression, and tumor re-growth in previously irradiated lesions in order to identify patients who will ultimately obtain clinical benefit from the systemic delivery of ICIs.

## Abbreviations

APCs, antigen-presenting cells; ATP, adenosine triphosphate; BBB, blood brain barrier; CTLA-4, cytotoxic T lymphocyte-associated antigen 4; CXCL10, CXC-motif chemokine ligand 10; DCs, dendritic cells; EVs, extracellular vesicles; IEXs, immune cell-derived exosomes; MDSCs, myeloid-derived suppressor cells; NSCLC, non-small cell lung cancer; OS, overall survival; PD-1, programmed cell death 1; PD-L1, programmed death-ligand 1; PFS, progression-free survival; PR, partial response; SD, stable disease; TAMs, tumor-associated macrophages; TEXs, tumor-derived exosomes; TGFβ, transforming growth factor beta; treg cells, Regulatory T cells
